# Diagnostic performance of four SARS-CoV-2 antibody assays in patients with COVID-19 or with bacterial and non-SARS-CoV-2 viral respiratory infections

**DOI:** 10.1007/s10096-021-04285-4

**Published:** 2021-06-09

**Authors:** Timo Huber, Philipp Steininger, Pascal Irrgang, Klaus Korn, Matthias Tenbusch, Katharina Diesch, Susanne Achenbach, Andreas E. Kremer, Marissa Werblow, Marcel Vetter, Christian Bogdan, Jürgen Held

**Affiliations:** 1 Mikrobiologisches Institut—Klinische Mikrobiologie, Immunologie und Hygiene, Universitätsklinikum Erlangen und Friedrich-Alexander-Universität (FAU) Erlangen-Nürnberg, Wasserturmstr. 3/5, 91054 Erlangen, Germany; 2 Virologisches Institut—Klinische und Molekulare Virologie, Universitätsklinikum Erlangen und Friedrich-Alexander-Universität (FAU) Erlangen-Nürnberg, Schlossgarten 4, 91054 Erlangen, Germany; 3 Center for Medical Information and Communication Technology, Universitätsklinikum Erlangen, Erlangen, Germany; 4 Transfusionsmedizinische und Hämostaseologische Abteilung, Universitätsklinikum Erlangen und Friedrich-Alexander-Universität (FAU) Erlangen-Nürnberg, Krankenhausstraße 12, 91054 Erlangen, Germany; 5 Department of Medicine 1, University Hospital Erlangen and Deutsches Zentrum Immuntherapie DZI, Friedrich-Alexander-University (FAU) Erlangen-Nürnberg, Ulmenweg 18, 91054 Erlangen, Germany; 6 Medical Immunology Campus Erlangen, Friedrich-Alexander University (FAU) Erlangen-Nürnberg, Schlossplatz 1, 91054 Erlangen, Germany

**Keywords:** COVID-19, SARS-CoV-2, Coronavirus, Antibody, Bacterial infections, Euroimmun, Vircell

## Abstract

SARS-CoV-2 antibody assays are used for epidemiological studies and for the assessment of vaccine responses in highly vulnerable patients. So far, data on cross-reactivity of SARS-CoV-2 antibody assays is limited. Here, we compared four enzyme-linked immunosorbent assays (ELISAs; Vircell SARS-CoV-2 IgM/IgA and IgG, Euroimmun SARS-CoV-2 IgA and IgG) for detection of anti-SARS-CoV-2 antibodies in 207 patients with COVID-19, 178 patients with serological evidence of different bacterial infections, 107 patients with confirmed viral respiratory disease, and 80 controls from the pre-COVID-19 era. In COVID-19 patients, the assays showed highest sensitivity in week 3 (Vircell-IgM/A and Euroimmun-IgA: 78.9% each) and after week 7 (Vircell-IgG: 97.9%; Euroimmun-IgG: 92.1%). The antibody indices were higher in patients with fatal disease. In general, IgM/IgA assays had only limited or no benefit over IgG assays. In patients with non-SARS-CoV-2 respiratory infections, IgG assays were more specific than IgM/IgA assays, and bacterial infections were associated with more false-positive results than viral infections. The specificities in bacterial and viral infections were 68.0 and 81.3% (Vircell-IgM/IgA), 84.8 and 96.3% (Euroimmun-IgA), 97.8 and 86.0% (Vircell-IgG), and 97.8 and 99.1% (Euroimmun-IgG), respectively. Sera from patients positive for antibodies against *Mycoplasma pneumoniae*, *Chlamydia psittaci*, and *Legionella pneumophila* yielded particularly high rates of unspecific false-positive results in the IgM/IgA assays, which was revealed by applying a highly specific flow-cytometric assay using HEK 293 T cells expressing the SARS-CoV-2 spike protein. Positive results obtained with anti-SARS-CoV-2 IgM/IgA ELISAs require careful interpretation, especially if there is evidence for prior bacterial respiratory infections.

**Supplementary Information:**

The online version contains supplementary material available at 10.1007/s10096-021-04285-4.

## Introduction

Coronavirus disease 2019 (COVID-19) is caused by the severe acute respiratory syndrome coronavirus 2 (SARS-CoV-2) which was first described in late December 2019 in Wuhan, China. Since then, it has become a pandemic infecting over 160 million people and resulting in approximately 3.3 million deaths worldwide [1].

Various direct and indirect assays have been developed to diagnose COVID-19. The mainstay for the diagnosis of acute infections is the detection of SARS-CoV-2 RNA and, to a lesser extent, of SARS-CoV-2 antigen in respiratory samples [2–4]. In contrast, the measurement of anti-SARS-CoV-2 antibodies in serum and plasma of patients is only of limited relevance for the early diagnosis because the sensitivity during the first 7 days after the onset of symptoms is on average 30% (95% confidence interval (CI) 21.4 to 40.7). This sensitivity increases to 70% (95% CI 63.5 to 79.5) during week 2 and to over 90% (95% CI 90.6 to 98.3) after week 3 [5]. Consequently, anti-SARS-CoV-2 antibody assays are mainly suitable for the retrospective diagnosis of previous COVID-19 infections in order to corroborate PCR results, to assess disease epidemiology or to test for potential SARS-CoV-2 immunity [2, 3]. The latter has received increasing attention since the start of the COVID-19 vaccination campaign. Serologic assays could be used to monitor vaccine-induced humoral immune responses in highly vulnerable (e.g., immunosuppressed) patients or to identify patients with prior (asymptomatic) COVID-19 infections, whose vaccination could be postponed as long as vaccine supplies are limited.

In this context, the specificity of anti-SARS-CoV-2 antibody assays is of particular importance because false-positive results could misleadingly suggest immunity. Reasons for such false-positive results are non-specific antibodies that cross-react with the target antigen, as well as components in the specimens (e.g., rheumatoid factor) that interfere with the assays [6]. Cross-reacting antibodies may be directed against closely related pathogens, like SARS-CoV-1, Middle East respiratory syndrome coronavirus (MERS-CoV), or seasonal human coronaviruses (e.g., HCoV-NL63, -229E, -HKU1), but sometimes also against viruses or bacteria that apparently have nothing in common with the target pathogen. For example, false-positive IgM results for Epstein-Barr virus (EBV) and cytomegalovirus (CMV) may occur in approximately 3% of patients with acute human immunodeficiency virus (HIV) infection and in 30% of patients with acute hepatitis A infection [7]. In addition, IgM antibodies generated after Parvovirus B19 infection were reported to cross-react with *Borrelia* spp., *Salmonella* spp., and *Campylobacter* spp. [8]. Previous studies with several thousand COVID-19 patients have shown that the specificity of serological assays may reach or even exceed 98% (95% CI 97.2 to 99.4) for IgG, IgM, IgA, IgG/IgM, and IgA/IgG combinations and total antibodies, respectively [5]. However, false-positive results are still possible and poorly understood, because data on cross-reactivity and interference with non-SARS-CoV-2 viral and bacterial respiratory infections is limited. Therefore, we initiated a study on patients with such infections using archived serum samples from the pre-COVID-19 era. Patients with and without COVID-19 served as controls.

## Materials and methods

Between May and December 2020, we conducted a retrospective cohort study at the University Hospital Erlangen (UKER), Germany, a 1400-bed tertiary care center. Serum or plasma samples of the following 4 patient groups were analyzed: group 1—patients with PCR-confirmed COVID-19 disease (COVID-19 group), group 2—patients with antibodies against bacterial respiratory pathogens (bacterial infection group), group 3—patients with previous PCR-confirmed respiratory viral disease (viral infection group), and group 4—randomly selected patients from 2017 (control group). The specimens of groups 2 to 4 were collected between January 2015 and September 2019, i.e., during the pre-COVID-19 era.

In the COVID-19 group, serum or plasma samples from inpatients of the UKER with detection of SARS-CoV-2 in nasopharyngeal swabs or respiratory specimens between March and June 2020 and from healthy blood donors with previous SARS-CoV-2 infection were included. The date of the first symptoms (disease onset), quantitative SARS-CoV-2 PCR results, and disease severity were obtained from the University Hospital clinical information system. The patients were classified as having mild disease (no oxygen administration or COVID-19-specific therapy needed), moderate disease (oxygen and/or COVID-19-specific therapy administered), severe disease (intensive care unit [ICU] treatment, artificial respiration), and fatal disease (death attributed to COVID-19).

The serum samples for the second and third groups were retrieved from the biobank of the Microbiology Institute of the UKER, where they had been kept frozen at − 20 °C. This biobank comprises all serum samples with pathologic results from the last decade and all serum samples with a request for fungal antigen measurement. The samples were selected as follows: In the bacterial infection group, all archived serum samples from the pre-COVID-19 era with antibody results indicating acute or previous infection with *Bordetella pertussis*, *Chlamydia psittaci*, *Chlamydia pneumoniae*, *Coxiella burnetii*, *Legionella pneumophila*, or *Mycoplasma pneumoniae* were included. In the viral infection group, all archived serum samples from the pre-COVID-19 era from patients with detection of respiratory viruses by PCR (influenza A/B virus, parainfluenza 1–4 virus, parechovirus, respiratory syncytial virus (RSV), adenovirus, enterovirus, rhinovirus, human metapneumovirus (HMPV), coronavirus, bocavirus) were included. As the multiplex PCR for respiratory viral infections also detects *Mycoplasma pneumoniae*-DNA, *Mycoplasma pneumoniae* was also included in the viral infection group. Patients below the age of 2, with hypoimmunoglobulinemia or with leukopenia, were excluded due to their (potential) impairment of antibody production. In addition, sera from the first 2 weeks after detection of a respiratory virus were excluded to allow for the generation and presence of potentially cross-reacting antibodies. In addition, 80 serum samples from 2017 were randomly selected as control group for the bacterial and viral infection groups.

The samples were tested for anti-SARS-CoV-2 antibodies with the COVID‐19 ELISA IgG and IgM/A (Vircell S.L., Granada, Spain) and with the anti-SARS-CoV-2-ELISA IgG and IgA (Euroimmun AG, Lübeck, Germany) according to the manufacturers’ instructions. Sera for IgM measurement were inactivated for 30 min at 56 °C. The Vircell assays use recombinant spike glycoprotein and nucleocapsid protein of SARS-CoV-2 as coated antigens, whereas the Euroimmun assays use only the spike glycoprotein. Both assays were performed on automated platforms (Vircell ThunderBolt^®^ and Euroimmun Analyzer I, respectively). The results were automatically calculated as antibody index (Vircell) and ratio (Euroimmun). For simplicity, we will further use the term antibody index for both assays. The cutoff values were as follows: Vircell-IgG (< 4 negative; 4–6 equivocal; > 6 positive), Vircell-IgM/A (< 6 negative; 6–8 equivocal; > 8 positive), and Euroimmun-IgG and Euroimmun-IgA (< 0.8 negative; 0.8–1.1 equivocal; ≥ 1.1 positive). All samples with equivocal results were retested and the mean of the two test runs was used as the reported result. Positive sera of the bacterial infection group were reassessed with a highly specific flow-cytometric assay using HEK 293 T cells expressing SARS-CoV-2 spike protein on their surface as described [9].

The study was approved by the ethics committee of the Friedrich Alexander University of Erlangen-Nürnberg (application number 174_20 B). All specimens were anonymized and the need for informed consent was waived.

Statistical analysis was performed using SPSS-V24 (IBM Corp., USA) and MedCalc-V19 (MedCalc Software Ltd., Belgium). Means were given with standard deviation (SD) and medians with interquartile range (IQR) in brackets. For comparison of means, the Mann–Whitney U test and the Kruskal–Wallis test were used. For comparison of categorical variables, the McNemar test or Fisher’s exact test was used. Pearson’s correlation coefficient was determined for linear correlation of two variables, and the effect size was interpreted according to Cohen [10]. p-values < 0.05 were considered significant differences.

## Results

An overview of the study groups is provided in Table 1.

**Table 1:** Overview of the study groups

	Number of patients	Positive serology or PCR	% of total patients (number)
COVID-19 group	207	SARS-CoV-2 SARS-CoV-2 with date of symptom onset SARS-CoV-2 with disease severity	100 (207) 90.8 (188) 100 (207)
Bacterial infection group	178	*Mycoplasma pneumoniae* *Chlamydia pneumoniae* *Bordetella pertussis* *Coxiella burnetii* *Legionella pneumophila* *Chlamydia psittaci*	41.5 (74) 18.6 (33) 13.5 (24) 13.5 (24) 9.6 (17) 3.9 (7)
Viral infection group	107	Rhinovirus Influenza virus A Parainfluenza virus Adenovirus Coronavirus OC43 Respiratory syncytial virus (RSV) Influenza virus B Bocavirus Coronavirus 229E Enterovirus Human metapneumovirus (HMPV) Coronavirus NL63 *Mycoplasma pneumoniae*	31.8 (34) 10.3 (11) 9.3 (10) 8.4 (9) 7.5 (8) 6.5 (7) 5.6 (6) 3.7 (4) 3.7 (4) 2.8 (3) 2.8 (3) 1.9 (2) 5.6 (6)
Pre-COVID-19 control group	80	–	–

*PCR*, polymerase chain reaction; *COVID-19*, coronavirus disease 2019; *SARS-CoV-2*, severe acute respiratory syndrome coronavirus type 2

### COVID-19 group.

**Fig. 1:**
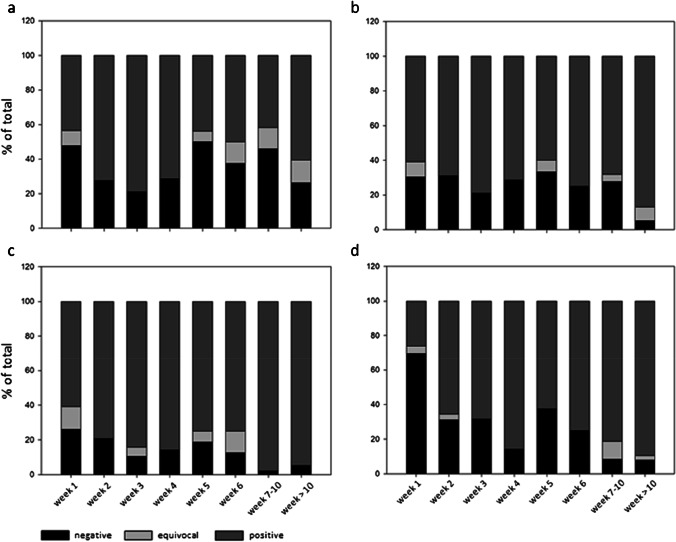
Categorical results of antibody indices against time after the onset of symptoms from the COVID-19 group. Black bars, light gray bars, or dark gray bars indicate a negative, equivocal, or positive test result, respectively. **a**) Vircell-IgM/A **b**) Euroimmun-IgA **c**) Vircell-IgG **d**) Euroimmun-IgG

A total of 345 serum or plasma samples from 207 patients with PCR-proven COVID-19 were tested (Table 2). When equivocal results were rated positive, the sensitivities of the assays were as follows: Vircell-IgM/A 68.4% (63.2–73.3), Euroimmun-IgA 77.1% (71.8–81.9), Vircell-IgG 87.8% (83.9–91.1), and Euroimmun-IgG 77.4% (72.6–81.7). The sensitivity of the Euroimmun-IgA and the Vircell-IgG assay were significantly higher (p < 0.01) than the sensitivity of the Vircell-IgM/A and the Euroimmun-IgG assay, respectively. For 188 patients, the exact dates of the onset of symptoms were available. The sensitivity of the Vircell-IgM/A and the Euroimmun-IgA assay was increasing from 52.2 and 69.6% in the first week to 78.9% for both assays in the third week. Afterwards, the sensitivities decreased to 54.2 and 72.3% in weeks seven to ten. In contrast, the sensitivity of the Vircell-IgG and the Euroimmun-IgG assay increased from 73.9 and 30.4% in the first week to 94.7% and 92.1 after week 10 (Fig. 1).

**Table 2:** SARS-CoV-2 antibody results in patients of the COVID-19 group

	Stratification	Vircell	Euroimmun	p-value	Vircell	Euroimmun	p-value
		IgM/A	IgA		IgG	IgG	
Sensitivity (equ results rated as pos) (%) (+ / − SD)	–	68.4 (63.2 − 73.3)	77.1 (71.8 − 81.9)	< 0.01	87.8 (83.9 − 91.1)	77.4 (72.6 − 81.7)	< 0.01
Sensitivity (equ results rated as neg) (%) (+ / − SD)	–	62.3 (57.0 − 67.5)	74.3 (68.7 − 79.3)	< 0.01	85.2 (81.0 − 88.8)	74.2 (69.2 − 78.7)	< 0.01
Sensitivity (equresults ratedas pos) (%)(+ / − SD)	First week	52.2 (30.9 − 73.2)	69.6 (47.1 − 86.8)	0.13	73.9 (51.6 − 89.8)	30.4 (13.2 − 52.9)	< 0.01
	Second week	72.4 (52.8 − 87.3)	69.0 (49.2 − 84.7)	1.00	79.3 (60.3 − 92.0)	69.0 (49.2 − 84.7)	0.25
	Third week	78.9 (54.4 − 93.9)	78.9 (54.4 − 93.9)	1.00	89.5 (66.9 − 98.7)	68.4 (43.5 − 87.4)	0.13
	Fourth week	71.4 (29.0 − 96.3)	71.4 (29.0 − 96.3)	1.00	85.7 (42.1 − 99.6)	85.7 (42.1 − 99.6)	1.00
	Fifth week	50.0 (24.7 − 75.3)	66.7 (38.4 − 88.2)	0.25	81.3 (54.4 − 96.0)	62.5 (35.4 − 84.8)	0.25
	Sixth week	62.5 (24.5 − 91.5)	75.0 (34.9 − 96.8)	1.00	87.5 (47.3 − 99.7)	75.0 (34.9 − 96.8)	1.00
	Seventh to tenthweek	54.2 (39.2 − 68.6)	72.3 (57.4 − 84.4)	0.049	97.9 (88.9 − 99.9)	91.7 (80.0 − 97.7)	0.25
	After tenth week	73.7 (56.9 − 86.6)	94.7 (82.3 − 99.4)	< 0.01	94.7 (89.3 − 99.6)	92.1 (78.6 − 98.3)	1.00
Sensitivity (equ results rated as neg) (%) (+ / − SD)	First week	43.5 (23.2 − 65.5)	60.9 (38.5 − 80.3)	0.13	60.9 (38.5 − 80.03)	26.1 (10.2 − 48.4)	< 0.01
	Second week	72.4 (52.8 − 87.3)	69.0 (49.2 − 84.7)	1.00	79.3 (60.3 − 92.0)	65.5 (45.7 − 82.1)	0.13
	Third week	78.9 (54.4 − 93.9)	78.9 (54.4 − 93.9)	1.00	84.2 (60.4 − 96.6)	68.4 (43.5 − 87.4)	0.25
	Fourth week	71.4 (29.0 − 96.3)	71.4 (29.0 − 96.3)	1.00	85.7 (42.1 − 99.6)	85.7 (42.1 − 99.6)	1.00
	Fifth week	43.8 (19.8 − 70.1)	60.0 (32.3 − 83.7)	0.25	75.0 (47.6 − 92.7)	62.5 (35.4 − 84.8)	0.50
	Sixth week	50.0 (15.7 − 84.3)	75.0 (34.9 − 96.8)	0.50	75.0 (34.9 − 96.8)	75.0 (34.9 − 96.8)	1.00
	Seventh to tenthweek	41.7 (27.6 − 56.8)	68.1 (52.9 − 80.9)	< 0.01	97.9 (88.9 − 99.9)	81.3 (67.4 − 91.1)	< 0.01
	After tenth week	60.5 (43.4 − 76.0)	86.8 (71.9 − 95.6)	0.01	94.7 (89.3 − 99.6)	89.5 (75.2 − 97.1)	0.63
Categorical result of SARS-CoV-2 antibody measurement (neg/equ/pos)	–	109/21/215	64/8/208	–	42/9/294	78/11/256	–
Categorical results, stratified according to the week after onset of symptoms (neg/equ/pos)	First week	11/2/10	7/2/14	–	6/3/14	16/1/6	–
	Second week	8/0/21	9/0/20		6/0/23	9/1/19	
	Third week	4/0/15	4/0/15		2/1/16	6/0/13	
	Fourth week	2/0/5	2/0/5		1/0/6	1/0/6	
	Fifth week	8/1/7	5/1/9		3/1/12	6/0/10	
	Sixth week	3/1/4	2/0/6		1/1/6	2/0/6	
	Seventh to tenthweek	22/6/20	13/2/32		1/0/47	4/5/39	
	After tenth week	10/5/23	2/3/33		2/0/36	3/1/34	
Categorical results, stratified according to disease severity (% pos) (neg/equ/pos)	Convalescent	58.9 (50/10/86)	79.8 (16/4/79)	< 0.01	95.2 (6/1/139)	85.6 (14/7/125)	< 0.01
	Mild	62.2 (31/6/61)	65.9 (28/2/58)	1.00	73.5 (22/4/72)	61.2 (36/2/60)	< 0.01
	Moderate	73.8 (15/1/45)	76.7 (13/1/46)	0.73	78.7 (10/3/48)	65.6 (20/1/40)	< 0.01
	Severe	58.8 (12/2/20)	74.0 (6/1/20)	0.69	88.2 (3/1/30)	76.5 (7/1/26)	0.13
	Fatal	50.0 (1/2/3)	83.3 (1/0/5)	1.00	83.3 (1/0/5)	83.3 (1/0/5)	1.00
Quantitative result of SARS-CoV-2 antibody measurement (median AI) (IQR)	Total	9.16 (3.92 − 20.82)	1.92 (0.67 − 4.21)	–	21.43 (7.58 − 38.42)	2.34 (0.43 − 4.26)	–
	Negative	3.44 (1.87 − 4.63)	0.39 (0.27 − 0.61)		2.33 (1.45 − 3.27)	0.20 (0.12 − 0.36)	
	Equivocal	6.89 (6.78 − 7.32)	1.05 (0.91 − 1.08)		4.97 (4.25 − 5.41)	0.98 (0.92 − 1.00)	
	Positive	21.7 (11.98 − 45.24)	3.22 (1.85 − 5.97)		33.78 (17.29 − 49.84)	4.15 (2.59 − 6.64)	
Quantitative results, stratified according to the week after onset of symptoms (median AI) (IQR)	First week	7.25 (3.50 − 16.41)	1.70 (0.53 − 3.88)	–	8.95 (3.92 − 19.32)	0.30 (0.17 − 1.25)	–
	Second week	47.71 (4.99 − 54.21)	1.78 (0.35 − 4.87)		24.52 (6.80 − 32.65)	2.46 (0.26 − 6.31)	
	Third week	28.26 (15.40 − 53.89)	2.63 (0.32 − 4.40)		32.49 (15.98 − 52.40)	4.33 (0.35 − 6.89)	
	Fourth week	24.13 (6.38 − 35.25)	1.27 (0.54 − 3.41)		29.21 (17.50 − 33.33)	2.96 (1.47 − 6.29)	
	Fifth week	5.89 (2.09 − 20.47)	1.11 (0.61 − 5.53)		17.06 (5.89 − 35.63)	2.34 (0.62 − 5.42)	
	Sixth week	9.43 (5.06 − 19.36)	2.47 (1.11 − 3.83)		20.82 (10.73 − 51.56)	3.29 (1.20 − 5.87)	
	Seventh to tenthweek	6.53 (3.38 − 11.74)	1.49 (0.71 − 2.82)		29.39 (13.34 − 46.12)	2.79 (1.61 − 4.35)	
	After tenth week	9.39 (5.68 − 12.09)	1.83 (1.19 − 3.69)		24.34 (11.89 − 41.84)	3.73 (2.57 − 4.94)	
Quantitative results, stratified according to disease severity (median AI) (IQR)	Convalescent	9.18 (4.91 − 15.18)	1.94 (1.16 − 3.73)	–	30.16 (15.92 − 47.54)	3.21 (1.70 − 4.94)	–
	Mild	11.08 (3.81 − 43.04)	1.55 (0.43 − 3.80)		20.08 (5.35 − 45.50)	1.99 (0.26 − 5.64)	
	Moderate	27.77 (7.15 − 48.88)	2.29 (0.35 − 4.21)		25.78 (8.03 − 43.21)	3.71 (0.22 − 7.07)	
	Severe	13.55 (4.21 − 25.31)	2.32 (0.87 − 6.32)		30.80 (7.96 − 50.54)	2.73 (1.12 − 6.67)	
	Fatal	23.19 (7.25 − 29.23)	6.69 (6.19 − 6.74)		37.33 (26.58 − 50.41)	5.54 (4.83 − 6.11)	

The p-values were determined for the comparison of the Vircell-IgM/A with the Euroimmun-IgA and the Vircell-IgG with the Euroimmun-IgG. *STD*, standard deviation; *IQR*, interquartile range; *neg*, negative; *equ*, equivocal; *pos*, positive; *AI*, antibody index

**Fig. 2:**
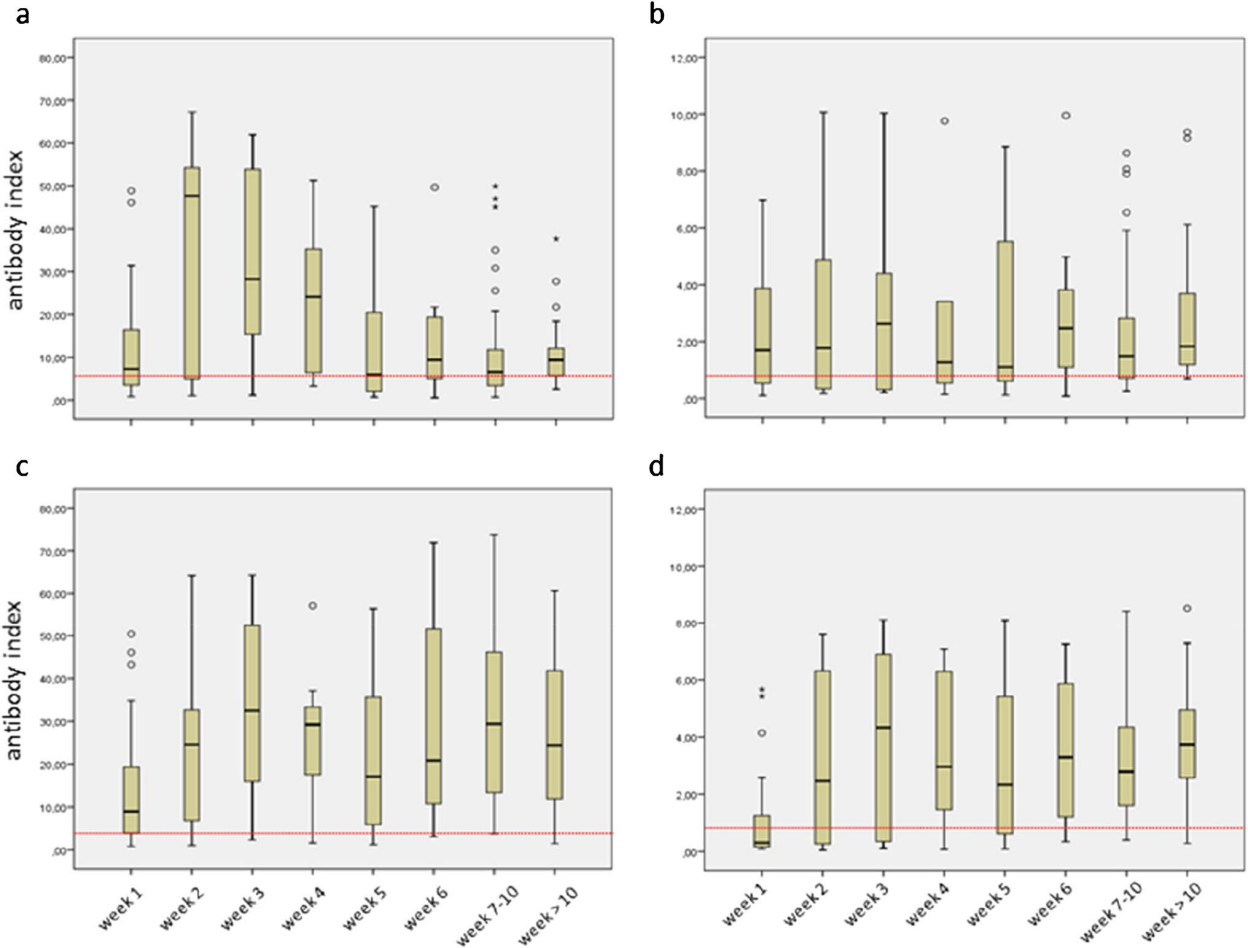
Box-plots of anti-SARS-CoV-2 antibody indices against time after the onset of symptoms from the COVID-19 group. The red dotted line indicates the lower manufacturer’s cutoff value (equivocal results are rated as positive results). Circles and stars depict outliers and extreme outliers, respectively. **a**) Vircell-IgM/A **b**) Euroimmun-IgA **c**) Vircell-IgG **d**) Euroimmun-IgG

The highest median antibody index was observed for Vircell-IgM/A in week 2 (47.7), for Euroimmun-IgA in week 3 (2.6), and for Vircell-IgG and Euroimmun-IgG in week 3 (32.4 and 4.3, respectively) (Fig. 2 and Fig. S1). Afterwards the median antibody indices of Vircell-IgM/A and Euroimmun-IgA decreased strongly to 9.3 and 1.8 at the end of the study period. In contrast, Vircell- and Euroimmun-IgG median antibody indices declined only moderately to 24.3 and 3.7, respectively. There was a strong correlation (large effect size) between the antibody indices of the Vircell-IgM/A and Euroimmun-IgA (r = 0.515, p < 0.01, n = 229) as well as the Vircell- and Euroimmun-IgG (r = 0.670, p < 0.01, n = 345) (Fig. S2).

**Fig. 3:**
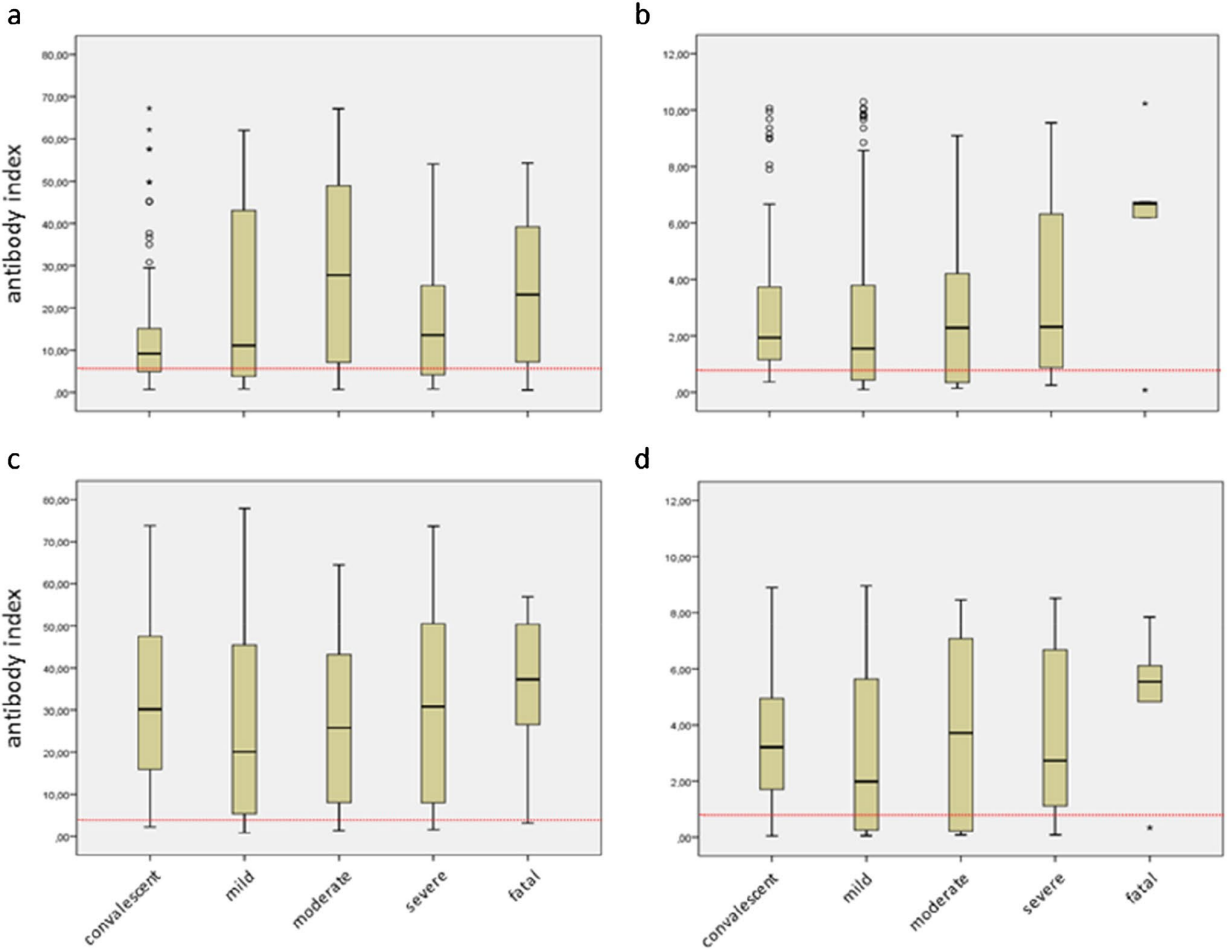
Box-plots of anti-SARS-CoV-2 antibody indices against disease severity from the COVID-19 group. The red dotted line indicates the lower manufacturer’s cutoff value (equivocal results are rated as positive results). Circles and stars depict outliers and extreme outliers, respectively. **a**) Vircell-IgM/A **b**) Euroimmun-IgA **c**) Vircell-IgG **d**) Euroimmun-IgG

Stratification of results according to disease severity revealed the highest median antibody indices for Vircell-IgM/A in patients with moderate (27.7) and fatal disease (23.1) and for Euroimmun-IgA, Vircell-IgG, and Euroimmun-IgG in fatal disease (6.7, 37.3, and 5.5, respectively) (Fig. 3). However, none of the antibody indices in patients with fatal course of disease was significantly elevated (Table 5).

### Bacterial infection group.

A total of 178 serum samples with antibody levels indicating bacterial respiratory infection were included (Table 3). When equivocal results were rated positive, the specificity of the assays were as follows: Vircell-IgM/A 68.0% (60.6–74.8), Euroimmun-IgA 84.8% (78.7–89.8), and Vircell-IgG and Euroimmun-IgG 97.8% (94.3–99.4) each. The specificity of the Vircell-IgM/A assay was significantly lower than the one of the Euroimmun-IgA assay (p < 0.01), which was mainly due to a high number of false-positive results obtained with sera from patients after *Mycoplasma pneumoniae* infections (n = 28 versus n = 11).

**Table 3:** SARS-CoV-2 antibody results in patients of the bacterial infection group

	Stratification	Vircell	Euroimmun	p-value	Vircell	Euroimmun	p-value
		IgM/A	IgA		IgG	IgG	
Specificity, (equ results rated as pos) (%) (+ / − SD)	–	68.0 (60.6 − 74.8)	84.8 (78.7 − 89.8)	< 0.01	97.8 (94.3 − 99.4)	97.8 (94.3 − 99.4)	1.00
Specificity, (equ results rated as neg) (%) (+ / − SD)	–	73.6 (66.5 − 79.9)	89.3 (83.8 − 93.5)	< 0.01	98.3 (95.2 − 99.7)	98.3 (95.2 − 99.7)	1.00
Categorical result of SARS-CoV-2 antibody measurement (% of patients) (number)	Negative Equivocal Positive	68.0 (121) 5.6 (10) 26.4 (47)	84.8 (151) 4.5 (8) 10.7 (19)	–	97.8 (174) 0.6 (1) 1.7 (3)	97.8 (174) 0.6 (1) 1.7 (3)	–
Categorical result stratified according to pathogen (% pos) (neg/equ/pos)	*Mycoplasma pneumoniae* *Chlamydia pneumoniae* *Bordetella pertussis* *Coxiella burnetii* *Legionella pneumophila* *Chlamydia psittaci*	38.9 (39/5/28) 12.5 (26/2/4) 8.3 (22/0/2) 12.5 (20/1/3) 31.3 (10/1/5) 57.1 (2/1/4)	15.3 (53/8/11) 6.3 (30/0/2) 8.3 (22/0/2) 4.2 (23/0/1) 18.8 (13/0/3) 0 (7/0/0)	0.01 0.29 1.00 0.38 0.38 –	1.4 (71/0/1) 3.1 (31/0/1) 0 (24/0/0) 0 (24/0/0) 6.3 (15/0/1) 0 (7/0/0)	1.4 (71/0/1) 3.1 (31/0/1) 0 (24/0/0) 4.2 (22/1/1) 0 (16/0/0) 0 (7/0/0)	1.00 1.00 – – – –
Quantitative result of SARS-CoV-2 antibody measurement (median AI) (IQR)	Total Negative Equivocal Positive	4.84 (2.96 − 8.47) 3.55 (2.47 − 4.91) 6.84 (6.30 − 7.05) 12.74 (9.80 − 26.20)	0.34 (0.19 − 0.56) 0.29 (0.19 − 0.43) 0.95 (0.89 − 1.00) 1.42 (1.22 − 1.75)	–	1.34 (0.96 − 2.27) 1.32 (0.93 − 2.23) 5.00 (–) 10.52 (8.75 − 14.28)	0.10 (0.07 − 0.13) 0.10 (0.07 − 0.13) 0.93 (–) 1.55 (1.37 − 2.12)	–
Quantitative results stratified according to pathogen (median AI) (IQR)	*Mycoplasma pneumoniae* *Chlamydia pneumoniae* *Bordetella pertussis* *Coxiella burnetii* *Legionella pneumophila* *Chlamydia psittaci*	5.84 (3.98 − 9.80) 3.30 (1.84 − 5.77) 4.03 (2.63 − 5.56) 3.50 (2.44 − 5.14) 4.65 (3.30 − 10.78) 10.66 (6.76 − 42.28)	0.46 (0.29 − 0.86) 0.27 (0.18 − 0.39) 0.30 (0.19 − 0.46) 0.20 (0.15 − 0.37) 0.42 (0.28 − 0.69) 0.24 (0.15 − 0.37)	–	1.42 (1.04 − 2.04) 1.39 (0.89 − 2.62) 1.38 (0.95 − 2.48) 1.13 (0.73 − 2.49) 1.14 (0.85 − 2.85) 1.26 (1.16 − 1.65)	0.12 (0.08 − 0.19) 0.09 (0.07 − 0.11) 0.07 (0.05 − 0.11) 0.10 (0.06 − 0.12) 0.09 (0.08 − 0.11) 0.09 (0.07 − 0.12)	–

The p-values were determined for the comparison of the Vircell-IgM/A with the Euroimmun-IgA and the Vircell-IgG with the Euroimmun-IgG. *STD*, standard deviation; *IQR*, interquartile range; *neg*, negative; *equ*, equivocal; *pos*, positive; *AI*, antibody index

**Fig. 4:**
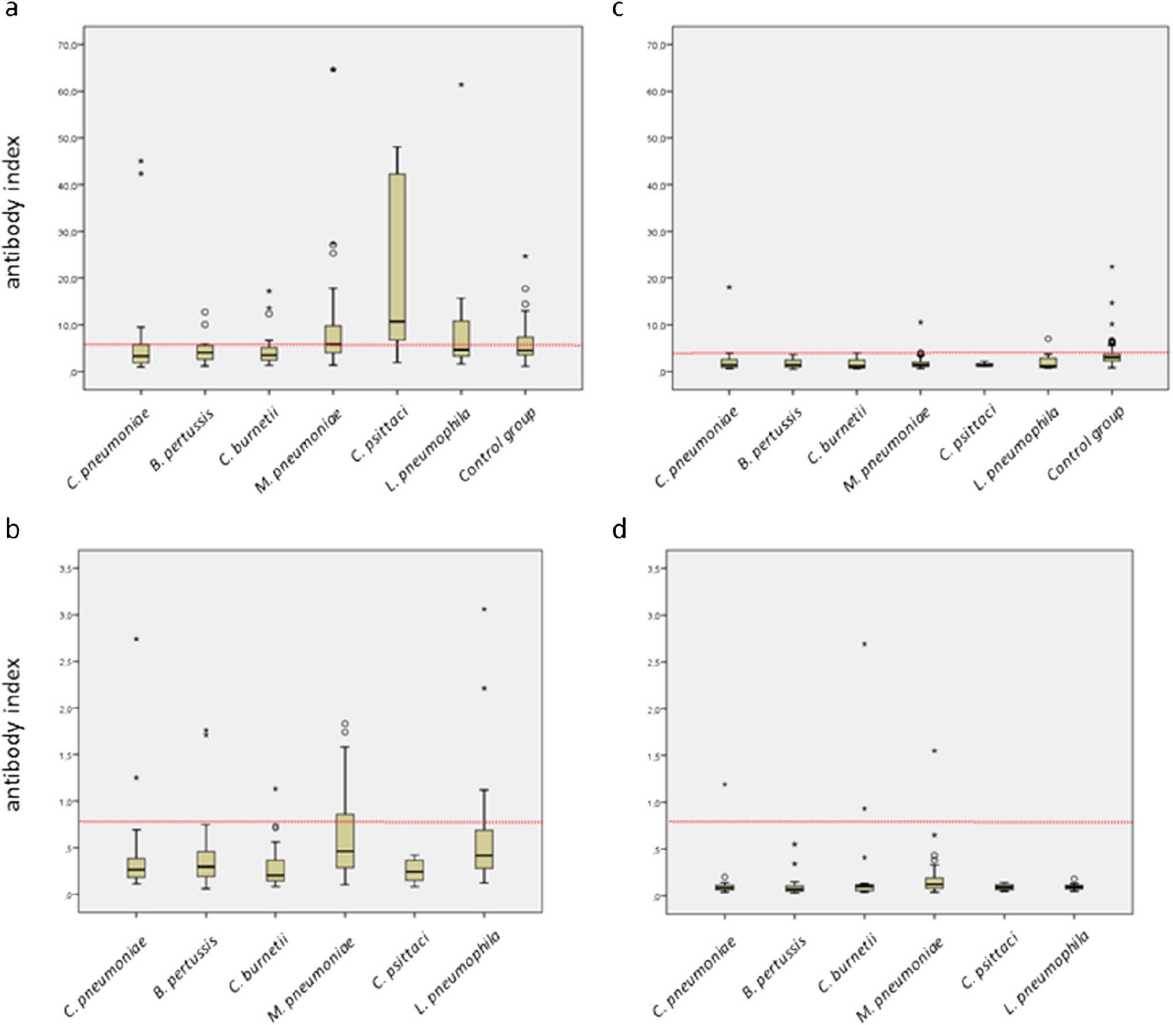
Box-plots of anti-SARS-CoV-2 antibody indices from the bacterial infection group and the control group. The red dotted line indicates the lower manufacturer’s cutoff value (equivocal results are rated positive results). Circles and stars depict outliers and extreme outliers, respectively. **a**) Vircell-IgM/A **b**) Euroimmun-IgA **c**) Vircell-IgG **d**) Euroimmun-IgG

A relevant elevation of the median antibody index was observed for certain bacterial pathogens with the Vircell-IgM/A assay only. In contrast, the median antibody indices of the Euroimmun-IgA and even more of the Vircell- and Euroimmun-IgG were considerably below the cutoff values of the assays. The highest median antibody indices of the Vircell-IgM/A assay were observed for *Chlamydia psittaci* (10.6), *Mycoplasma pneumoniae* (5.8), and *Legionella pneumophila* (4.6) (Fig. 4). The differences were statistically significant for *Chlamydia psittaci* (p = 0.02) and *Mycoplasma pneumoniae* (p < 0.01). Although elevated, the median antibody index of positive samples in the bacterial infection group was still significantly lower than the one of the COVID-19 group for all assays (Vircell-IgM/A: 12.7 versus 21.7; Vircell-IgG: 10.5 versus 33.8; Euroimmun-IgA: 1.4 versus 3.2; Euroimmun-IgG: 1.6 versus 4.1).

All positive and equivocal samples of the bacterial infection group were retested with a highly specific flow-cytometric assay using HEK 293 T cells expressing SARS-CoV-2 spike protein on their surface. Using the IgM version of this assay, none of the 47 positive and 10 equivocal results of the Vircell-IgM/A assay and none of the 19 positive and 8 equivocal results of the Euroimmun-IgA assay was confirmed. One of three positive results in the Vircell-IgG assay and one of three positive results in the Euroimmun-IgG assay were weakly positive in the flow-cytometric IgG assay.

### Viral infection group.

A total of 107 serum samples of patients with recent infection with respiratory viruses or *Mycoplasma pneumoniae* were included (Table 4). When equivocal results were rated as positive, the specificities of the assays were as follows: Vircell-IgM/A 81.3% (72.6–88.2), Euroimmun-IgA 96.3% (90.7–99.0), Vircell-IgG 86.0% (77.9–91.9), and Euroimmun-IgG 99.1% (94.9–100.0). The specificity of the Vircell assays was significantly lower than the one of the corresponding Euroimmun assays (p < 0.01).

**Table 4:** SARS-CoV-2 antibody results in patients of the viral infection group

	Stratification	Vircell	Euroimmun	p-value	Vircell	Euroimmun	p-value
		IgM/A	IgA		IgG	IgG	
Specificity, (equ results rated as pos) (%) (+ / − SD)	–	81.3 (72.6 − 88.2)	96.3 (90.7 − 99.0)	< 0.01	86.0 (77.9 − 91.9)	99.1 (94.9 − 100.0)	< 0.01
Specificity, (equ results rated as neg) (%) (+ / − SD)	–	87.9 (80.1 − 93.4)	96.3 (90.7 − 99.0)	0.02	95.3 (89.4 − 98.5)	100.0 (96.6 − 100.0)	-
Categorical result of SARS-CoV-2 antibody measurement (% of patients) (number)	Negative Equivocal Positive	81.3 (87) 6.5 (7) 12.1 (13)	96,3 (103) 0 (0) 3.7 (4)	-	86.0 (92) 9.3 (10) 4.7 (5)	99.1 (106) 0.9 (1) 0 (0)	-
Categorical results of SARS-CoV-2 antibody measurement stratified according to pathogen (% pos) (neg/equ/pos)	Adenovirus Bocavirus Coronavirus • Coronavirus 229E • Coronavirus NL63 • Coronavirus OC43 Enterovirus Human metapneumovirus Influenza virus A Influenza virus B Parainfluenza virus Rhinovirus Respiratory syncytial virus *Mycoplasma pneumoniae*	11.1 (8/0/1) 25 (2/1/1) 15.4 (11/1/2) 25 (2/1/1) 50 (1/0/1) 0 (8/0/0) 0 (3/0/0) 33.3 (2/0/1) 18.2 (8/1/2) 16.7 (4/1/1) 10 (9/0/1) 8.8 (28/3/3) 0 (7/0/0) 16.7 (5/0/1)	0 (9/0/0) 0 (4/0/0) 0 (14/0/0) 0 (4/0/0) 0 (2/0/0) 0 (8/0/0) 33.3 (2/0/1) 0 (3/0/0) 9.1 (10/0/1) 16.7 (5/0/1) 0 (10/0/0) 2.9 (33/0/1) 0 (7/0/0) 0 (6/0/0)	- - - - - - - - - 1.00 - 0.13 - -	0 (8/1/0) 0 (4/0/0) 7.1 (12/1/1) 25 (3/0/1) 0 (2/0/0) 0 (7/1/0) 0 (3/0/0) 0 (3/0/0) 0 (9/2/0) 0 (6/0/0) 10 (9/0/1) 8.8 (26/5/3) 0 (6/1/0) 0 (6/0/0)	0 (9/0/0) 0 (4/0/0) 0 (10/0/0) 0 (4/0/0) 0 (4/0/0) 0 (2/0/0) 0 (8/0/0) 0 (3/0/0) 0 (11/0/0) 0 (6/0/0) 0 (10/0/0) 0 (33/1/0) 0 (7/0/0) 0 (6/0/0)	- - - - - - - - - - - 0.04 - -
Quantitative result of SARS-CoV-2 antibody measurement (median AI) (IQR)	Total Negative Equivocal Positive	3.23 (1.91 − 5.28) 2.67 (1.50 − 4.10) 7.17 (6.72 − 7.35) 9.70 (9.35 − 12.76)	0.20 (0.10 − 0.33) 0.19 (0.10 − 0.30) - 2.67 (1.60 − 4.43)	-	1.89 (1.23 − 3.13) 1.68 (1.18 − 2.51) 4.83 (4.50 − 5.04) 7.16 (6.58 − 7.61)	0.07 (0.05 − 0.11) 0.07 (0.05 − 0.11) 1.05 (-) -	-
Quantitative results stratified according to pathogen (median AI) (IQR)	Adenovirus Bocavirus Coronavirus • Coronavirus 229E • Coronavirus NL63 • Coronavirus OC43 Enterovirus Human metapneumovirus Influenza virus A Influenza virus B Parainfluenza virus Rhinovirus Respiratory syncytial virus *Mycoplasma pneumoniae*	2.28 (0.82 − 3.03) 5.92 (3.87 − 8.15) 2.87 (2.01 − 7.34) 6.30 (4.23 − 8.35) 13.90 (2.29 − 25.50) 2.32 (1.00 − 3.46) 3.23 (3.15 − 3.57) 5.23 (4.94 − 7.29) 3.67 (1.59 − 6.42) 4.08 (1.66 − 7.97) 2.16 (1.48 − 4.55) 3.63 (2.20 − 5.17) 2.10 (1.40 − 3.43) 4.72 (4.08 − 5.76)	0.21 (0.07 − 0.25) 0.41 (0.28 − 0.47) 0.12 (0.07 − 0.35) 0.51 (0.41 − 0.57) 0.12 (0.09 − 0.14) 0.08 (0.05 − 0.29) 0.28 (0.26 − 1.79) 0.11 (0.10 − 0.17) 0.22 (0.10 − 0.34) 0.22 (0.13 − 0.30) 0.10 (0.15 − 0.37) 0.18 (0.10 − 0.34) 0.11 (0.09 − 0.21) 0.21 (0.14 − 0.28)	-	1.31 (0.98 − 1.46) 3.25 (2.53 − 3.60) 2.09 (1.26 − 2.80) 2.76 (2.16 − 5.21) 2.02 (1.64 − 2.40) 1.52 (0.93 − 2.85) 1.95 (1.35 − 2.44) 3.70 (3.00 − 3.80) 1.69 (1.31 − 3.21) 1.37 (1.05 − 1.73) 1.92 (1.57 − 3.42) 2.26 (1.26 − 3.59) 1.52 (1.07 − 1.89) 2.42 (1.82 − 2.97)	0.08 (0.03 − 0.10) 0.08 (0.05 − 0.11) 0.06 (0.04 − 0.09) 0.07 (0.05 − 0.10) 0.07 (0.04 − 0.09) 0.06 (0.05 − 0.10) 0.10 (0.09 − 0.11) 0.05 (0.05 − 0.06) 0.08 (0.07 − 0.11) 0.06 (0.05 − 0.07) 0.08 (0.06 − 0.12) 0.07 (0.05 − 0.14) 0.06 (0.05 − 0.07) 0.09 (0.05 − 0.14)	-

The p-values were determined for the comparison of the Vircell-IgM/A with the Euroimmun-IgA and the Vircell-IgG with the Euroimmun-IgG. *STD*, standard deviation; *IQR*, interquartile range; *neg*, negative; *equ*, equivocal; *pos*, positive; *AI*, antibody index

False-positive results were most common when using the Vircell-IgM/A ELISA. The highest percentage of Vircell-IgM/A false-positive results was observed with sera from patients infected with human bocavirus (50%, n_POS_ = 2), HMPV (33.3% n_POS_ = 1), and influenza B virus (33.3%, n_POS_ = 2). However, sample numbers in these groups were very small, with the false-positive rates being based on one or two positive samples only. Sera of patients infected with non-SARS-CoV-2 coronaviruses showed a positivity rate of only 15.4% in the Vircell-IgM/A ELISA. Interestingly, 50% of serum samples of Coronavirus NL63–infected and Coronavirus 229E–infected patients tested positive in this ELISA, but again these results were based on single positive samples.

The highest median antibody index of Vircell-IgM/A was measured in sera of patients infected with human bocavirus (5.9), HMPV (5.2), or *Mycoplasma pneumoniae* (4.7). Again, the group of non-SARS-CoV-2 coronaviruses showed a low median antibody index (2.9), but Coronavirus NL63 (13.9) and Coronavirus 229E (6.3) antibody indices were high. Comparison of means showed a trend towards higher Vircell-IgM/A antibody indices for samples from patients with Coronavirus NL63 (p = 0.08) or *Mycoplasma pneumoniae* (p = 0.07) infections. The median antibody index of Coronavirus 229E (0.51) was the highest with the Euroimmun-IgA assay, and it significantly exceeded that of the other viral pathogens (p < 0.01).

### Control group.

**Fig. 5:**
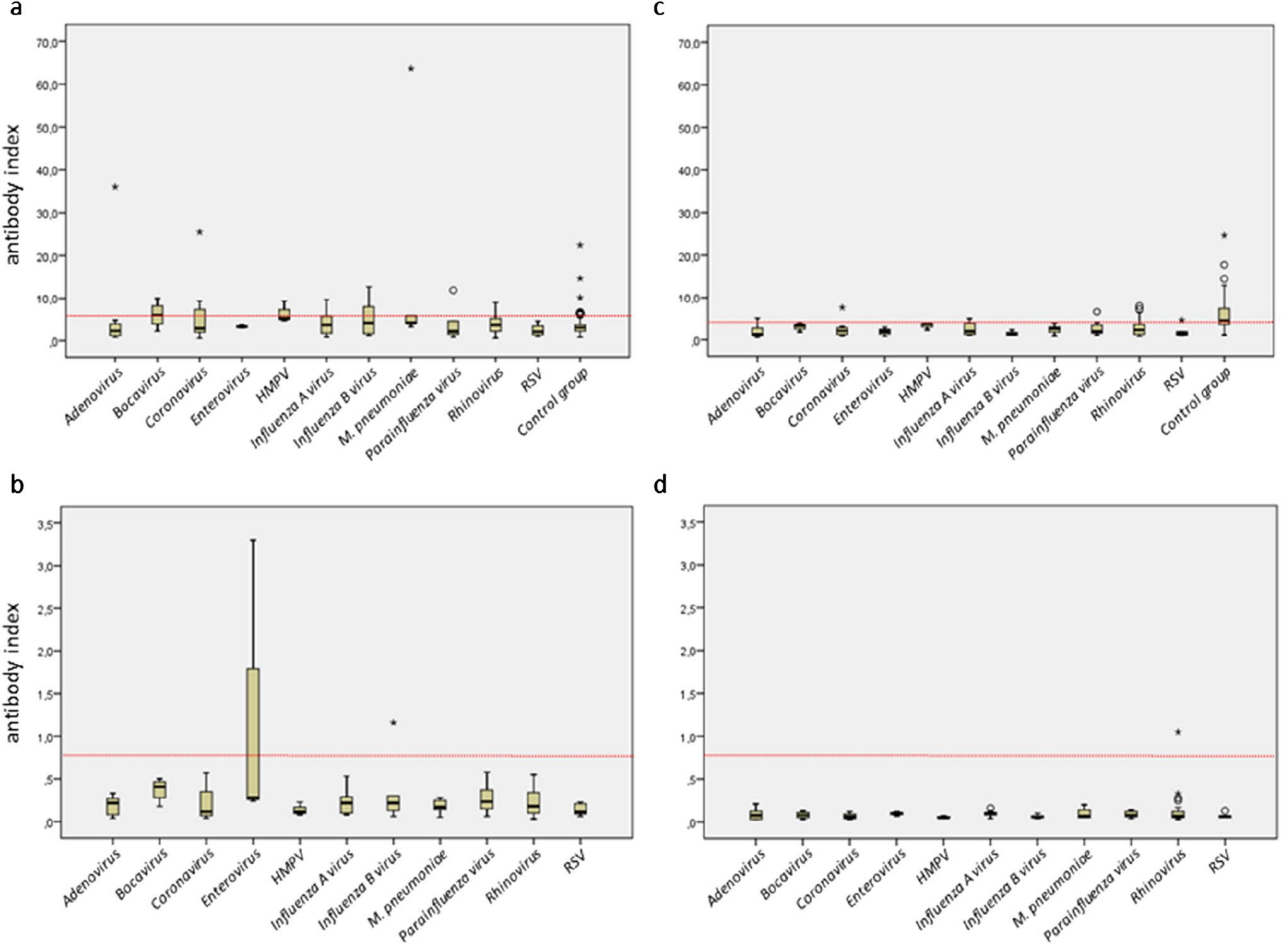
Box-plots of anti-SARS-CoV-2 antibody indices from the viral infection group and the control group. The red dotted line indicates the lower manufacturer’s cutoff value (equivocal results are rated positive results). Circles and stars depict outliers and extreme outliers, respectively. **a**) Vircell-IgM/A **b**) Euroimmun-IgA **c**) Vircell-IgG **d**) Euroimmun-IgG

A total of 80 randomly selected serum samples of patients from 2017 served as pre-COVID-19 control group for the Vircell assays. The control group was not measured with the Euroimmun assays because of their high specificity. All results are presented in Table 5 and in Figs. 4 and 5. When equivocal results were rated as positive, the specificities were as follows: Vircell-IgM/A 72.5% (61.4–81.9) and Vircell-IgG 86.0% (77.9–91.9). The median antibody indices of Vircell-IgM/A and Vircell-IgG were 4.5 (3.5–7.4) and 3.0 (2.6–3.7), respectively.

**Table 5:** SARS-CoV-2 antibody results in patients of the pre-COVID era control group

	Stratification	Vircell		Comparison with the bacterial infection group		Comparison with the viral infection group	
		IgM/A	IgG	Vircell-IgM/A	Vircell-IgG	Vircell-IgM/A	Vircell-IgG
Specificity, (equ results rated as pos) (%) (+ / − SD)	-	72.5 (61.4 − 81.9)	81.3 (80.0 − 89.1)	p = 0.56	p < 0.01	0.16	0.42
Specificity, (equ results rated as neg) (%) (+ / − SD)	-	75.0 (64.1 − 84.0)	90.0 (81.2 − 95.6)	p = 0.88	p < 0.01	0.03	0.24
Categorical result of SARS-CoV-2 antibody measurement (number) (neg/equ/pos)	-	58/2/20	65/7/8	-	-	-	-
Quantitative result of SARS-CoV-2 antibody measurement (median AI) (IQR)	Total Negative Equivocal Positive	4.52 (3.55 − 7.40) 3.75 (3.20 − 4.76) 6.66 (6.60 − 6.71) 10.18 (9.17 − 12.22)	3.03 (2.56 − 3.69) 2.73 (2.15 − 3.21) 5.16 (4.58 − 5.55) 6.60 (6.30 − 12.42)	p = 0.84	p < 0.01	p < 0.01	p < 0.01

*STD*, standard deviation; *IQR*, interquartile range; *neg*, negative; *equ*, equivocal; *pos*, positive; *AI*, antibody index

### Receiver operating characteristic analysis.

**Fig. 6:**
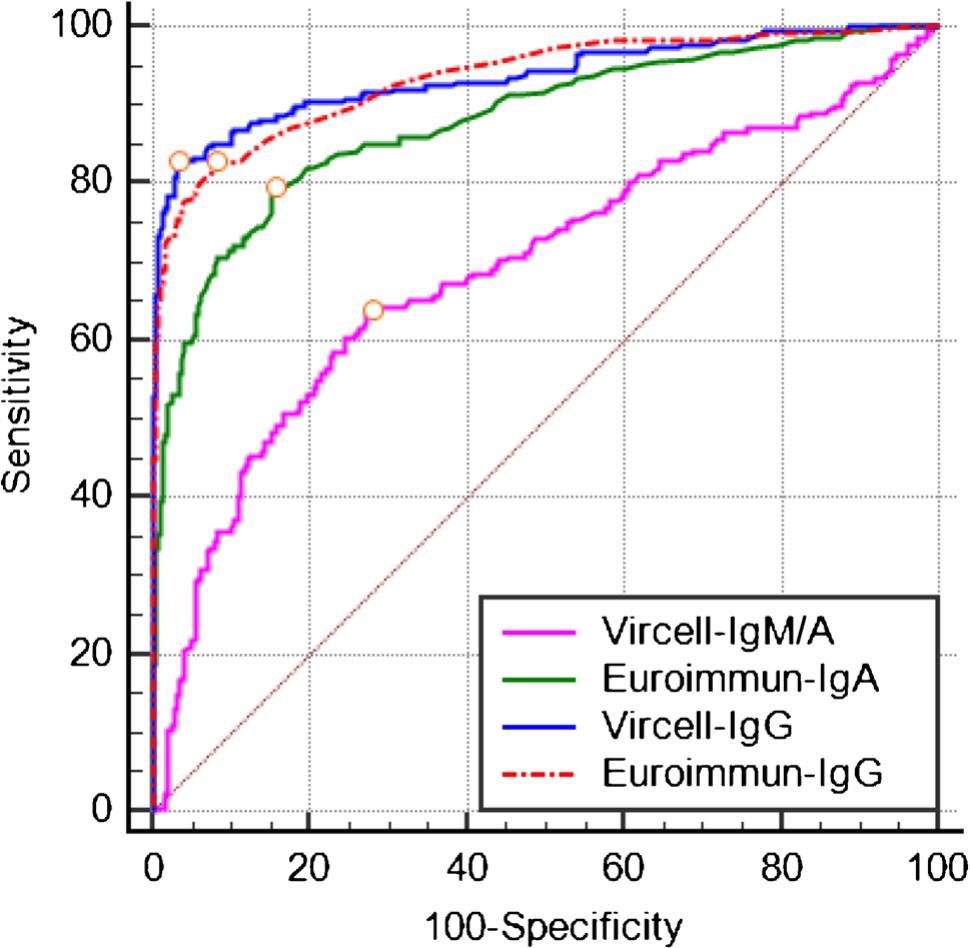
ROC curves of the four anti-SARS-CoV-2 antibody assays (COVID-19 group and pre-COVID-19 control group). The AUC of the Vircell-IgG and the Euroimmun-IgG was not significantly different (p = 0.82), but the AUC of both assays was significantly higher than the AUC of the Vircell-IgM/A and Euroimmun-IgA assay (p < 0.01). The circles depict the highest Youden index

ROC analysis of all results from the COVID-19 group and the control group gave areas under the ROC curve (AUC) of 0.693 (0.651–0.733) for Vircell-IgM/A, 0.880 (0.849–0.907) for Euroimmun-IgA, 0.937 (0.913 to 0.957) for Vircell-IgG, and 0.935 (0.910 to 0.955) for Euroimmun-IgG (Fig. 6). The AUC values of the Vircell-IgG and the Euroimmun-IgG were not significantly different from each other (p = 0.82), but significantly higher than the AUC of the Vircell-IgM/A and Euroimmun-IgA assay (p < 0.01). The cutoffs with the highest Youden index were 6.7 for Vircell-IgM/A (sensitivity 67.0%, specificity 75.3%), 0.6 for Euroimmun-IgA (sensitivity 79.5%, specificity 84.6%), 6.6 for Vircell-IgG (sensitivity 83.8%, specificity 97.3%), and 0.3 for Euroimmun-IgG (sensitivity 86.1%, specificity 94.0%).

## Discussion

In the present study, we evaluated the diagnostic performance of four anti-SARS-CoV-2 antibody assays from two manufacturers using serum samples of patients with COVID-19 or with bacterial or viral respiratory infections acquired prior to the COVID-19 era.

Both the Vircell and the Euroimmun assays are enzyme-linked immunosorbent assays (ELISAs). However, the Vircell assays use recombinant spike glycoprotein and nucleocapsid protein of SARS-CoV-2 as coated antigens, whereas the Euroimmun assays use only the spike glycoprotein.

In patients of the COVID-19 group (Figs. 1 and 2), our data is in line with the classical viral response pattern, where IgM is the first antibody class to appear, followed closely by IgA and by long lasting IgG. Considering the test sensitivities, the Vircell-IgM/A and Euroimmun-IgA assay started with values between 50 and 70% in week 1 and reached a maximum around 80% in week 3. In contrast, the Vircell-IgG and the Euroimmun-IgG sensitivities were increasing from 70 and 30%, respectively, in week 1 to over 90% at the end of the study period. Interestingly, the Vircell-IgG was superior to the Vircell-IgM/A in terms of sensitivity throughout the entire study period. The same was true for the Euroimmun-IgG assay, except for week 1, when the Euroimmun-IgA assay was more sensitive. Thus, there was no or only limited advantage in using the IgM/A assays as compared to that in using the IgG assays.

Our data point to a correlation between disease severity and the antibody index level, as also suggested by recently published studies [11, 12]. Patients with fatal disease had particularly high antibody indices, especially when using the Euroimmun assays. However, these results have to be interpreted with caution, because the number of patients with fatal disease was limited (n = 5) so that none of the antibody indices of patients with fatal disease was significantly elevated. Furthermore, the observed increase of antibody indices with severity of disease might reflect the different time-points of serum sampling. Sera of patients with mild and moderate disease originated mainly from the first 3 weeks after the onset of symptoms, whereas sera of patients with severe disease were from weeks five to ten. As shown in the present study, notably, IgG antibody indices were higher at the end of the study period.

Our study confirms previous data that showed increasing sensitivities of SARS-CoV-2 antibody assays until week 3 [5]. In addition, our data demonstrated that the sensitivities stayed high until week 10 and beyond. However, neither the IgM/A assays nor the IgG assays showed sensitivities higher than 90% for the diagnosis of COVID-19 during the first 6 weeks of disease. Therefore, antibody assays can only serve as adjunctive tests in diagnosing early COVID-19. Instead, they are best used either during the late phase of infection, when PCR may have become negative, or for the retrospective diagnosis of previous SARS-CoV-2 infections and for ascertaining immunity in convalescent patients.

The pre-COVID-19 bacterial and viral infection groups and the control group were analyzed to determine the specificity of the antibody assays. While the specificities of the IgG assays in general were very high (95–100%), the IgM/A assays showed numerous false-positive results. The Vircell-IgM/A assay had a specificity in the bacterial and viral infection group of approximately 70% and 85%, respectively, which was significantly lower than the values obtained with the Euroimmun-IgA assay in both groups.

The low IgM/A specificity in the bacterial infection group was mostly due to false-positive results with sera from patients with antibodies against *Chlamydia psittaci*, *Mycoplasma pneumoniae*, and *Legionella pneumophila*. Comparison of the Vircell-IgM/A antibody index of sera from patients positive for antibodies against *Chlamydia psittaci* or *Mycoplasma pneumoniae* with the pre-COVID-19 control group showed that in both cases, antibody indices were significantly elevated (p = 0.02). *Mycoplasma pneumoniae* exerts potent mitogenic effects on B cells leading to polyclonal antibody synthesis [13–15]. Furthermore, antibodies synthesized by mitogenically activated B cells are most often of the IgM class. Consequently, the increase in serum lgM after *Mycoplasma pneumoniae* infection is only partly due to pathogen-specific antibodies [13]. Cold agglutinins and a variety of autoimmune antibodies against cardiolipins, lung, brain, and smooth muscle antigens are produced [15]. Thus, it is not surprising that sera of patients with *Mycoplasma pneumoniae* infection showed false-positive results in other antibody assays [16, 17]. This is in line with our observation that no cross-reactivity between *Mycoplasma pneumoniae* and SARS-CoV-2 was seen with the highly specific flow-cytometric assay using HEK 293 T cells expressing SARS-CoV-2 spike protein on their surface, suggesting non-specific interactions in the Vircell-IgM/A assay. In addition, the manufacturer described interference of the Vircell-IgM/A assay with antinuclear antibodies [18]. Interestingly, *Mycoplasma*-induced false-positive SARS-CoV-2 IgM results have already led to misdiagnoses. Serrano et al. recently reported *Mycoplasma pneumoniae*-induced skin lesions in two patients that were misdiagnosed as COVID-19-associated skin disease based on false-positive SARS-CoV-2 IgM results [19].

The median antibody indices in the viral infection group were all below the manufacturers’ cutoff values. Therefore, previous viral infections did not influence the specificities of the assays as prominently as certain bacterial infections. Especially, the Euroimmun assays exhibited an excellent specificity when evaluated with sera from patients with other viral infections. Interestingly, sera from patients with previous Coronavirus NL63 or Coronavirus 229E infection showed high median antibody indices in the Vircell-IgM/A assay, but less so in the Euroimmun-IgA assay. However, because of the limited number of patients diagnosed with these pathogens, more data are necessary to confirm our observations.

Previous analyses on cross-reactivity with non-SARS-CoV-2 pathogens yielded conflicting results. Some studies reported no cross-reactivity with various viruses, bacteria, and fungi at all [20, 21], while others experienced false-positive results in sera of patients with SARS-CoV-1 or MERS-Coronavirus [22]; Coronavirus 229E, NL63, OC43, HKU1, or SARS-CoV-1 [23]; CMV [24]; influenza A and B virus, adenovirus, or *Mycoplasma pneumoniae* [25]. One study used the Euroimmun-IgA and IgG assays [26]. Lassauniere *et al.* examined acute viral respiratory tract infections with other coronaviruses (n = 5) or non-coronaviruses (n = 45); dengue virus (n = 9), CMV (n = 2), and EBV (n = 10). The Euroimmun-IgA cross-reacted primarily with adenovirus and influenza A/B and to a lesser extent with EBV and dengue virus, whereas the Euroimmun-IgG cross-reacted with Coronavirus HKU1 and adenovirus [26]. The latter study is in line with our observation that false-positive results occur with the Euroimmun-IgA almost exclusively with sera from patients infected with influenza A/B and enterovirus and that the specificity of the Euroimmun-IgA assay was clearly lower than that of the Euroimmun-IgG assay.

A weakness of our study is that the diagnoses in the bacterial infection group were based on serologic assays and that PCR confirmation was performed only in a minority of patients. Therefore, false-positive serology results are possible.

In conclusion, the Vircell- and the Euroimmun-IgG assays are superior to the Vircell-IgM/A and Euroimmun-IgA assays. Both IgG assays showed very good sensitivities more than 7 weeks after infection and excellent specificities. Therefore, they are suitable for diagnosing previous COVID-19 infection and for testing SARS-CoV-2 humoral immunity following immunization with a spike glycoprotein–based vaccine.

## Supplementary Information

Below is the link to the electronic supplementary material.

Supplementary file1 (PDF 348 KB)

## Data Availability

The original data sets of this study are available from the corresponding author (JH), upon reasonable request.

## References

[1] WHO Coronavirus (COVID-19) dashboard. World Health Organization. https://covid19.who.int/. Accessed 14 May 2021.

[2] Caruana G, Croxatto A, Coste AT, Opota O, Lamoth F, Jaton K, Greub G (2020). Diagnostic strategies for SARS-CoV-2 infection and interpretation of microbiological results. Clin Microbiol Infect.

[3] Theel ES, Slev P, Wheeler S, Couturier MR, Wong SJ, Kadkhoda K: The role of antibody testing for SARS-CoV-2: is there one? J Clin Microbiol 2020, 58(8). 10.1128/JCM.00797-20 PMC7383527 32350047

[4] Dinnes J, Deeks JJ, Adriano A, Berhane S, Davenport C, Dittrich S, Emperador D, Takwoingi Y, Cunningham J, Beese S et al: Rapid, point-of-care antigen and molecular-based tests for diagnosis of SARS-CoV-2 infection. Cochrane Database Syst Rev 2020, 8:CD013705. 10.1002/14651858.CD013705 PMC8078202 32845525

[5] Deeks JJ, Dinnes J, Takwoingi Y, Davenport C, Spijker R, Taylor-Phillips S, Adriano A, Beese S, Dretzke J, Ferrante di Ruffano L et al: Antibody tests for identification of current and past infection with SARS-CoV-2. Cochrane Database Syst Rev 2020, 6:CD013652. 10.1002/14651858.CD013652 PMC7387103 32584464

[6] Woods CR (2013). False-positive results for immunoglobulin M serologic results: explanations and examples. J Pediatric Infect Dis Soc.

[7] Post JJ, Chan MK, Whybin LR, Shi Q, Rawlinson WD, Cunningham P, Robertson PW (2011). Positive Epstein-Barr virus and cytomegalovirus IgM assays in primary HIV infection. J Med Virol.

[8] Tuuminen T, Hedman K, Soderlund-Venermo M, Seppala I (2011). Acute parvovirus B19 infection causes nonspecificity frequently in Borrelia and less often in Salmonella and Campylobacter serology, posing a problem in diagnosis of infectious arthropathy. Clin Vaccine Immunol.

[9] Lapuente D, Maier C, Irrgang P, Hubner J, Peter AS, Hoffmann M, Ensser A, Ziegler K, Winkler TH, Birkholz T et al: Rapid response flow cytometric assay for the detection of antibody responses to SARS-CoV-2. Eur J Clin Microbiol Infect Dis 2020. 10.1007/s10096-020-04072-7 PMC7572153 33078221

[10] Cohen J (1992). A power primer. Psychol Bull.

[11] Li K, Huang B, Wu M, Zhong A, Li L, Cai Y, Wang Z, Wu L, Zhu M, Li J (2020). Dynamic changes in anti-SARS-CoV-2 antibodies during SARS-CoV-2 infection and recovery from COVID-19. Nat Commun.

[12] Cervia C, Nilsson J, Zurbuchen Y, Valaperti A, Schreiner J, Wolfensberger A, Raeber ME, Adamo S, Weigang S, Emmenegger M *et al*: Systemic and mucosal antibody responses specific to SARS-CoV-2 during mild versus severe COVID-19. J Allergy Clin Immunol 2021, 147(2):545–557 e549. 10.1016/j.jaci.2020.10.040 PMC7677074 33221383

[13] Biberfeld G, Gronowicz E (1976). Mycoplasma pneumoniae is a polyclonal B-cell activator. Nature.

[14] Simecka JW, Ross SE, Cassell GH, Davis JK (1993). Interactions of mycoplasmas with B cells: antibody production and nonspecific effects. Clin Infect Dis.

[15] Ruuth E, Praz F (1989). Interactions between mycoplasmas and the immune system. Immunol Rev.

[16] Miyashita N, Akaike H, Teranishi H, Kawai Y, Ouchi K, Kato T, Hayashi T, Okimoto N (2013). Atypical Pathogen Study G: Chlamydophila pneumoniae serology: cross-reaction with Mycoplasma pneumoniae infection. J Infect Chemother.

[17] Montagnani F, Rossetti B, Vannoni A, Cusi MG, De Luca A (2018). Laboratory diagnosis of Mycoplasma pneumoniae infections: data analysis from clinical practice. New Microbiol.

[18] Vircell SL: COVID‐19 ELISA IgM+IgA manual. L‐MA1032‐EN‐01: Indirect immunoenzyme assay to test IgM+IgA antibodies against SARS‐CoV‐2 in human serum/plasma. REVISED: 2020‐04‐07. 2020.

[19] Monte Serrano J, Garcia-Gil MF, Cruanes Monferrer J, Aldea Manrique B, Prieto-Torres L, Garcia Garcia M, Matovelle Ochoa C, Ara-Martin M (2020). COVID-19 and Mycoplasma pneumoniae: SARS-CoV-2 false positive or coinfection?. Int J Dermatol.

[20] Jiehao C, Jin X, Daojiong L, Zhi Y, Lei X, Zhenghai Q, Yuehua Z, Hua Z, Ran J, Pengcheng L (2020). A case series of children with 2019 novel Coronavirus infection: clinical and epidemiological features. Clin Infect Dis.

[21] Xu K, Chen Y, Yuan J, Yi P, Ding C, Wu W, Li Y, Ni Q, Zou R, Li X (2020). Factors associated with prolonged viral RNA shedding in patients with Coronavirus Disease 2019 (COVID-19). Clin Infect Dis.

[22] Freeman B, Lester S, Mills L, Rasheed MAU, Moye S, Abiona O, Hutchinson GB, Morales-Betoulle M, Krapinunaya I, Gibbons A et al: Validation of a SARS-CoV-2 spike protein ELISA for use in contact investigations and serosurveillance. bioRxiv 2020.

[23] Guo L, Ren L, Yang S, Xiao M, Chang, Yang F, Dela Cruz CS, Wang Y, Wu C, Xiao Y et al: Profiling early humoral response to diagnose novel coronavirus disease (COVID-19). Clin Infect Dis 2020, 71(15):778–785. 10.1093/cid/ciaa310 PMC7184472 32198501

[24] Infantino M, Grossi V, Lari B, Bambi R, Perri A, Manneschi M, Terenzi G, Liotti I, Ciotta G, Taddei C (2020). Diagnostic accuracy of an automated chemiluminescent immunoassay for anti-SARS-CoV-2 IgM and IgG antibodies: an Italian experience. J Med Virol.

[25] Zhang J, Zhang X, Liu J, Ban Y, Li N, Wu Y, Liu Y, Ye R, Liu J, Li X et al: Serological detection of 2019-nCoV respond to the epidemic: a useful complement to nucleic acid testing. Int Immunopharmacol 2020, 88:106861. 10.1016/j.intimp.2020.106861 PMC7391978 32771946

[26] Lassaunière R, Frische A, Harboe ZB, Nielsen ACY, Fomsgaard A, Krogfelt KA, Jørgensen CS: Evaluation of nine commercial SARS-CoV-2 immunoassays. medRxiv 2020:2020.2004.2009.20056325.

