# Supplementary material for: Diagnostic performance of four SARS-CoV-2 antibody assays in patients with COVID-19 or with bacterial and non-SARS-CoV-2 viral respiratory infections
Source: Eur J Clin Microbiol Infect Dis. 2021 Jun 9;40(9):1983–97. doi: 10.1007/s10096-021-04285-4 (PMC8189710; doi:10.1007/s10096-021-04285-4)
Supplement: Supplementary file 1 — Supplementary file1 (PDF 348 KB) [file 10096_2021_4285_MOESM1_ESM.pdf]

Supplementary Information:

Diagnostic performance of four SARS-CoV-2 antibody assays in patients with COVID-19 or with bacterial and non-SARS-CoV-2 viral respiratory infections

Infection

Timo Huber, Philipp Steininger, Pascal Irrgang, Klaus Korn, Matthias Tenbusch, Katharina Diesch, Susanne Achenbach, Andreas E. Kremer, Marissa Werblow, Marcel Vetter, Christian Bogdan, Jürgen Held[#]

[#]Corresponding author: Jürgen Held, Mikrobiologisches Institut, Universitätsklinikum Erlangen
Wasserturmstr. 3/5, 91054 Erlangen, Germany
Email: juergen.held@uk-erlangen.de; Telephone: +49-9131 85 46903; Fax: +49-9131 85 22117

Table S1: Comparison of anti-SARS-CoV-2 antibody index means of the study groups

	**Vircell IgM/A**	**Euroimmun IgA**	**Vircell IgG**	**Euroimmun IgG**
Comparison of time after onset of symptoms [p-value]	<0.01	0.93	0.01	<0.01
Pairwise comparison of one time span against all others, [p-value]				
- First week	0.20	0.75	<0.01	<0.01
- Second week	<0.01	0.58	0.37	0.96
- Third week	<0.01	0.80	0.11	0.20
- Fourth week	0.41	0.73	0.89	0.72
- Fifth week	0.10	0.80	0.28	0.71
- Sixth week	0.84	0.66	0.75	0.59
- Seventh to tenth week	<0.01	0.49	0.04	0.43
- after tenth week	0.54	0.18	0.52	0.09
Comparison of severity of disease [p-value]	< 0.01	0.17	0.02	0.08
Pairwise comparison of one disease severity against all others, [p-value]				
- convalescent	<0.01	0.27	<0.01	0.19
- mild	0.56	0.11	<0.01	0.01
- moderate	<0.01	0.44	0.29	0.62
- severe	0.77	0.43	0.63	0.72
- fatal	0.70	0.09	0.50	0.13
Comparison of all bacterial pathogen groups [p-value]	< 0.01	< 0.01	0.85	< 0.01

Pairwise comparison of one bacterial pathogen against all others, [p-value]				
- *Mycoplasma pneumoniae* versus other bacterial pathogens	< 0.01	< 0.01	0.51	< 0.01
- *Chlamydia pneumoniae* versus other bacterial pathogens	< 0.01	0.04	0.61	0.11
- *Bordetella pertussis* versus other bacterial pathogens	0.09	0.31	0.98	0.01
- *Coxiella burnetii* versus other bacterial pathogens	0.04	<0.01	0.22	0.31
- *Legionella pneumophila* versus other bacterial pathogens	0.63	0.23	0.64	0.48
- *Chlamydia psittaci* versus other bacterial pathogens	0.02	0.13	0.90	0.55
Comparison of all viral pathogen groups [p-value]	0.06	0.07	0.31	0.85
Pairwise comparison of one viral pathogen against all others [p-value]				
- Adenovirus versus other viral pathogens	0.16	0.53	0.19	0.46
- Bocavirus versus other viral pathogens	0.14	0.08	0.15	0.99
- Coronavirus (229E+NL63+OC43) versus other viral pathogens	0.87	0.86	0.98	0.29
- Coronavirus 229E versus other viral pathogens	0.08	<0.01	0.16	0.82
- Coronavirus NL63 versus other viral pathogens	0.50	0.34	0.94	0.58
- Coronavirus OC43 versus other viral pathogens	0.06	0.10	0.31	0.38
- Enterovirus versus other viral pathogens	0.40	0.48	0.45	0.22
- HMPV versus other viral pathogens	0.10	0.29	0.17	0.10
- Influenza virus A versus other viral pathogens	0.94	0.84	0.95	0.44
- Influenza virus B versus other viral pathogens	0.57	0.77	0.11	0.21
- *Mycoplasma pneumoniae* versus other viral pathogens	0.07	0.87	0.64	0.44
- Parainfluenzavirus versus other viral pathogens	0.26	0.42	0.69	0.64
- Rhinovirus versus other viral pathogens	0.93	0.86	0.25	0.35
- RSV versus other viral pathogens	0.24	0.23	0.07	0.21

The p-values were determined for the comparison of categories within one antibody assay and not between different antibody assays. The mean antibody indices were compared between all categories/pathogen groups (Kruskal-Wallis test) and pairwise between one category/pathogen against all others (Mann-Whitney-U test). HMPV, human metapneumovirus; RSV, respiratory syncytial virus.

Table S2: Number of equivocal measurements and results after test repetition

**Study group**		**Vircell**		**Euroimmun**	
		**IgM/A**	**IgG**	**IgA**	**IgG**
**COVID-19 group**	Number of equivocal results after first test run	33	20	13	11
	Results of test repetition (negative/equivocal/positive)	5/21/7	5/9/6	0/8/5	0/11/0
**Bacterial infection group**	Number of equivocal results after first test run	24	7	11	1
	Results of test repetition (negative/equivocal/positive)	12/7/5	5/1/1	0/8/3	0/1/0

**Viral infection group**	Number of equivocal results after first test run	11	13	0	1
	Results of test repetition (negative/equivocal/positive)	4/5/2	0/9/4	---	0/1/0
**Pre-COVID-19 control group**	Number of equivocal results after first test run	13	13	---	---
	Results of test repetition (negative/equivocal/positive)	10/2/1	4/7/2	---	---
**total**	Number of equivocal results after first test run	81	53	24	13
	Results of test repetition (negative/equivocal/positive)	31/35/15	14/26/13	0/16/8	0/13/0

COVID-19, coronavirus disease 2019.

Fig. S1: Antibody indices from the COVID-19 group against the time after the onset of symptoms

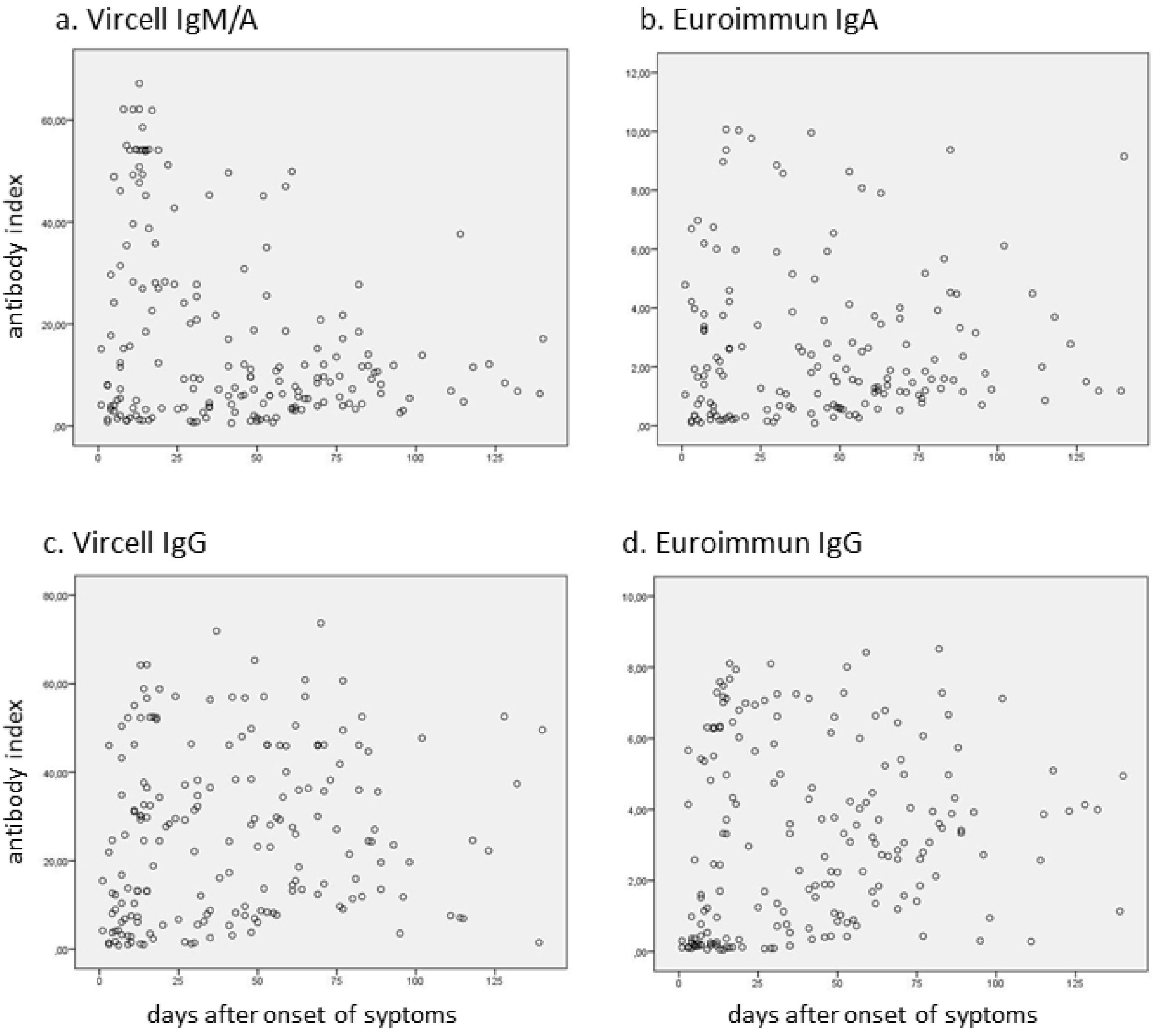

Fig. S2: Correlation of antibody indices between the Vircell and Euroimmun assays in the COVID-19 group

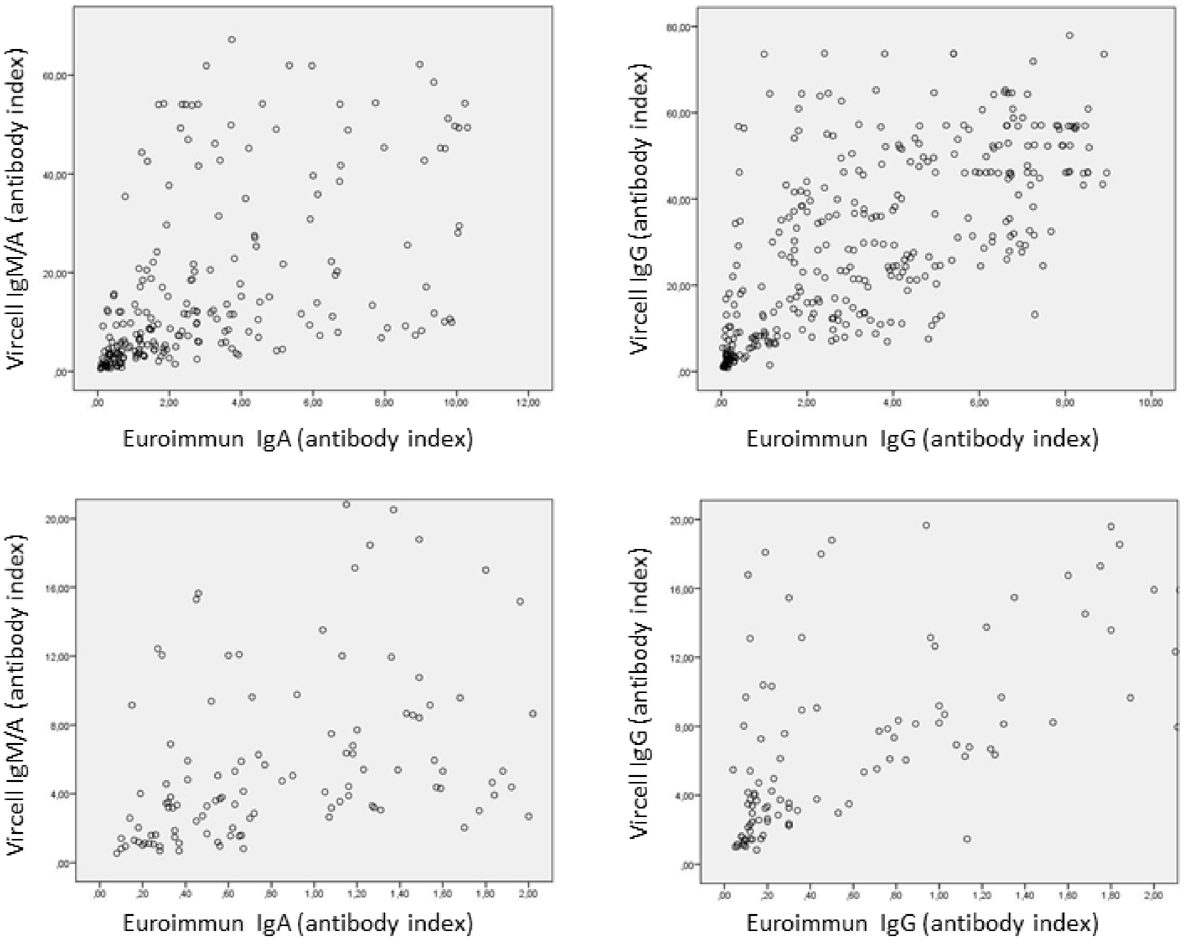

Antibody indices of the two anti-SARS-CoV-2 IgM/A assays or the two IgG assays were plotted against each other. The upper two scatter plots show all results whereas the lower two scatter plots focus on the samples with smaller antibody indices for better visualization. There was a strong correlation between the antibody indices of the Vircell-IgM/A and Euroimmun-IgA (r=0.515, p<0.01, n=229) as well as the Vircell- and Euroimmun-IgG (r=0.670, p<0.01, n=345). R, Pearson's correlation coefficient.
